# Surprisingness and Occupational Engagement Influence Affective Forecasting in Career-Relevant Contexts

**DOI:** 10.3389/fpsyg.2022.838765

**Published:** 2022-07-01

**Authors:** Di Lu, Runkai Jiao, Feifei Li, Xiaoqing Lin, Hang Yin

**Affiliations:** ^1^School of Psychology, Northeast Normal University, Changchun, China; ^2^National Training Center for Kindergarten Principals, Ministry of Education, Northeast Normal University, Changchun, China

**Keywords:** information accessibility, occupational engagement, surprisingness, career-related emotions, affective forecasting

## Abstract

People tend to misestimate their future emotions. This phenomenon is thought to be associated with information accessibility. However, few studies have demonstrated the impact of context-specific information accessibility on affective forecasting. This research investigated the effects of information accessibility on affective forecasting in career context (i.e., occupational engagement was seen as information accessibility), during which surprise or not surprise context was played simultaneously. We found that affective forecasting appeared stably across emotional response types. Specifically, there was an underestimation in interest appraisals and an overestimation in satisfaction. These biases were influenced by occupational engagement, which only worked in career interest appraisals. High occupational engagement made people estimate their future emotions more accurately and overcome their forecasting bias. Surprisingness was then manipulated further to explain whether it could impact the effect of occupational engagement on affective forecasting. The emotional responses in both prediction and experience were affected by surprisingness, thus causing no affective forecasting biases. These results suggest the role of occupational engagement in affective forecasting and provide evidence supporting the information accessibility model about the mechanism in affective forecasting.

## Introduction

People tend to confuse their future feelings and always overestimate or underestimate the intensity of future emotions (Gilbert et al., 1998; Wilson et al., 2000). The misestimate of emotional reactions to future events, known as impact bias, is a basic psychological phenomenon in affective forecasting (Wilson and Gilbert, 2003). Focalism and adaption neglect are always used to explain why this robust and pervasive misestimation exists. Focalism proposes that the central characteristics of future events are focused on, whereas other marginal information is ignored (Gilbert et al., 1998; Wilson et al., 2000). Relatively, by reminding people of their daily lives (marginal information) beyond sports competition (central characteristics), the daily record list can improve emotional prediction accuracy (Gilbert and Ebert, 2002). Adaption neglection proposes that predictions are made in a vacuum, leading to a typical exaggeration of future feelings (Diener et al., 2006). Thus, it cannot size up people’s adaptability when they overcome those extremes. For example, converging evidence shows that healthy people (compared with patients) tend to overestimate the negative emotional impact of deteriorated health conditions (e.g., detection of HIV and positive kidney transplants; for an overview, see Peters et al., 2014). Beyond these basic explanations of mixed findings regarding bias, some research tries to find a more general theory to explore the mechanism of affective forecasting. Furthermore, they believe that some distinct features of predicted emotion can be considered (e.g., Frank et al., 2020).

The information accessibility model suggests that information accessibility is a determinant of the shift from prediction to experience, and it influences affective forecasting as a distinct feature (Robinson and Clore, 2002). High information accessibility will overpredict the value sensitivity than those with low information accessibility. The general evaluability theory asserts that existing inner states (e.g., individual preference) are more vivid for decision-makers. It makes people base affective forecasting on the current affective state rather than the potential future state (Hsee and Zhang, 2010). These theories share a core assumption: information characteristics involving affective forecasting are the key. The more accurate is the token of events, the better is forecasting (Gilbert and Wilson, 2009). In other words, the best way to overcome affective forecasting bias is still to truly “feel” about it. For example, some studies have demonstrated that it is hard work for people in a “hot” emotional state to predict what they may want in “cold” (in the future) and *vice versa* (Schkade and Kahneman, 1998; Van Boven et al., 2000). These studies usually ask participants to predict their future feelings and then engage in these events immediately. However, most real-life events are of long-interval, making it hard to manipulate information accessibility during real decision-making. Furthermore, as affective forecasting bias relies on available information, relatively strict manipulations of information may be necessary. Understanding the real-life influence of information accessibility on affective forecasting both inside and outside the laboratory is crucial to discussing the mechanism of bias.

Specific to career context, occupational engagement can represent information accessibility, providing available information to fund a dual decision-making process *via* direct experience (Krieshok et al., 2009). It implies increasing awareness through experiential activities about the career world and themselves. As experiential learning, occupational engagement engenders a propensity to accumulate helpful experience and information, making decision-making optimal. More occupational engagement means planning more for the future. Findings suggest that occupational engagement is essential to participants’ career success and well-being (Scott, 2006; Cox, 2008). Decision-makers claim that they only prefer the rational process, but they rely on both rational and intuitive processes (Klein, 2004, 2017). Occupational engagement plays a critical role in judging which process is more available. It also encourages people to participate in those behaviors that help career decision-makers get acquainted with the larger world. Considering that people are unclear about their preferred career decision-making styles, it may represent real-life situations better to use occupational engagement to detect information accessibility (Krieshok et al., 2009). To date, only one study has examined the effect of occupational engagement on affective forecasting, where it is just referred to as an influencing factor on dual-process among college students (Motl et al., 2018).

The process of career decision-making, or “occupational engagement” here, is full of surprisingness induced by uncertainty. It refers to not only the engagement of the current occupation but also reactions to those other unpredictable chances and events beyond the job. Many researchers in the career field have also suggested this uncertainty and its effects (e.g., positive uncertainty, Gelatt, 1989; planned happenstance, Mitchell et al., 1999; complexity theory, Drodge, 2002). It is worth emphasizing that this surprise is also likely related to affective forecasting because it is bound to the duration of an emotional experience (Verduyn et al., 2009). Furthermore, the surprisingness of the to-be-predicted event (how much the event violates prior expectations) has been found relevant to the forecast bias in the effect of mood (Study 1, Lench et al., 2019). Lench et al. (2019) manipulate surprise in their lab study 5 through the likelihood of acceptance versus rejection for the paid task (all participants are ultimately rejected in the feedback). Specifically, participants in the low surprise condition are told that hardly anybody is accepted, and it is more likely that they will complete the alternative task. In the control condition, participants are told nothing about the likelihood of being chosen. And in the high surprise condition, people are told that almost everyone is accepted. Following the previous evidence (Verduyn et al., 2012, 2013), the surprise condition ought to have affected the degree of overestimation in forecasts. Results, however, provide a different pattern in this laboratory manipulation. People across conditions forecast their “ambiguous emotion” as if no surprise difference exists (i.e., surprise did not influence bias in forecasting). After some exploratory analyses across studies, researchers interpret this pattern as the difference in intensity of emotional responses. However, according to the differentiated model of affective forecasting (Lench et al., 2019), three cognitive features of emotions are independent and affect separate sections of affective forecasting, respectively. This premise makes their explanation less convincing.

Moreover, the high surprise condition, associated with high uncertainty, makes people feel more disgust and discomfort than the low surprise condition (e.g., Hogg, 2007). Therefore, the surprise may affect people’s affective forecasting bias *via* the emotional experience phase. The information accessibility model assumes that information is not treated independently but is not equally effective (e.g., Robinson and Clore, 2002; Hsee and Zhang, 2010). For example, researchers find that curiosity leads people to actively expose themselves to aversive stimuli without apparent benefits (Hsee and Ruan, 2016). And this irrational behavior exists just because people tend to resolve uncertainty, which touches their inherent desire independent of considerations for consequences. In other words, the tendency to resolve uncertainty is more effective at that moment, with more accessibility than any other information overwhelmingly. Following this principle, the dissociation between field study and lab study, according to Lench et al. (2019), has another explanation: people are likely to rely on the information with more attractiveness. In their field study, uncertainty is more vivid and attractive and thus more effective for people. If so, the surprise may influence affective forecasting when the manipulated situation is more counterfactual. Furthermore, if providing some other information with more accessibility, the influence of surprise would be missing.

### Overview of Experiments

This study conducted two experiments to test the above hypotheses. In Experiment 1, we explored whether occupational engagement affected participants’ emotional responses across time. Moreover, we explored whether this effect would differ among career decision-making processes across two frequent emotions (i.e., interest appraisals or satisfaction). We choose these two emotions because they are typical representatives of occupational emotions. Previous investigations revealed that some discrete emotions also show affective forecasting errors (regret, Buchanan et al., 2019; guilt and shame, van Dijk et al., 2017). Given the difference between discrete emotions in neurophysiological processes and social functions (Colombetti and Giovanna, 2009), a precise understanding of how people forecast their occupational emotions rather than general emotions is needed to learn about occupational affective forecasting. Based on its findings, in Experiment 2, we then examined whether the events’ surprisingness would affect the relationship between occupational engagement and emotional responses. Specifically, we investigated the differences between participants’ forecasts and actual feelings about occupational events so as to explore the effects of occupational engagement and surprise on affective forecasting. Experiment 1 focused on a truly career environment (i.e., participants work as kindergarten principals), and participants were assigned to either group (high vs. low) by their occupational engagement levels. In contrast, Experiment 2 focused on an experimental manipulation (i.e., providing students with occupational/educational videos), and participants were also randomly assigned to various surprisingness groups (expected vs. unexpected) by pre-decisional manipulation (in addition to grouping by occupational engagement levels).

In general, we predicted that occupational engagement would influence affective forecasting in both emotions. Also, surprise could affect this affective forecasting bias. According to adaptive career decision-making theory (Krieshok et al., 2009; Motl et al., 2018), the greater the occupational engagement was (more available information), the less likely participants made a huge forecasting bias. In contrast, the less occupational engagement people possessed, the more likely they predicted worse. Given the dissociation of mechanisms between affective forecasting under field study and lab study (e.g., Lench et al., 2019), the pattern of surprise on affective forecasting in our study was predicted to be complicated: surprise, in isolation, did affect bias. Nevertheless, with occupational engagement playing an assistant, the surprise would not influence affective forecasting.

## Experiment 1: Effect of Occupational Engagement on Kindergarten Principals’ Career Interest Appraisals and Satisfaction

Experiment 1 explored whether occupational engagement influenced participants’ emotional responses across time in a truly career environment (i.e., working as kindergarten principals). As this research is part of a big data collection project funded by the Chinese Ministry of Education about the improvement of the ability of kindergarten principals, we chose kindergarten principals as representatives of staff members who have engaged in jobs and possess some occupational experience. In Experiment 1, Chinese kindergarten principals were instructed to forecast and report their actual feeling about their career events during the first month of the Autumn term. This experiment aimed to investigate patterns of affective forecasting bias in a career context through different types of emotions. We selected career interest appraisals and career satisfaction as dependent variables to reflect forecasting biases and included occupational engagement as a primary impact factor.

### Methods

#### Participants

This experiment adopted a mixed design, in which participants were assigned to either group by their occupational engagement levels (high vs. low). *A priori* power analysis indicated that 86 participants were essential for repeat measurement analysis to have 80% power for detecting a medium effect with the traditional α = 0.05 criterion of statistical significance (G*Power 3.1: Faul et al., 2007). Thus, a total of 271 Chinese kindergarten principals first completed OES-W (*Occupational Engagement Scale-Worker*, for more details refer to the “Materials” section). We then assigned 63 participants to the high-occupational-engagement group with *z*-scores greater than + 0.5 and 56 participants to the low-occupational-engagement group with *z*-scores lower than −0.5, separately. Three participants (one from the high group and two from the low group) were found not to participate in Time 2 (Experience) measure. As a result, a total of 116 participant cases remained for the formal analysis (110 females, *M*_age_ = 47.41, *SD* = 5.05). An independent-samples *t*-test confirmed that the grouping was successful, and participants in high-engagement condition would have more occupational engagement (*N* = 62, *M* = 49.63, *SD* = 0.49) than participants in low-engagement condition (*N* = 54, *M* = 39.96, *SD* = 6.08), *t*(115) = −11.64, *p* < 0.001, *d* = 2.24. Moreover, all participants signed a written, informed consent under the procedures and protocols approved by the human subjects review committee of Northeast Normal University.

#### Materials

##### Occupational Engagement Scale-Worker

The OES-W is a 20-item instrument to measure workers’ career-related experience validated by Scott (2006), and it has been proven reliable (α = 0.83). It contained two dimensions of occupational engagement, namely, one named *job involvement*, focus on jumping in one’s current position and being involved in; the other labeled as *job curiosity*, focus on keeping an eye on different jobs and curious about what is beyond the present. And for the particular occupation of participants in this experiment (principals, supreme governor of one kindergarten), only the *job involvement* was investigated. Internal consistency of the OES-W in the current sample (α = 0.93) suggests sufficient reliability.

##### Measurements for Interest and Satisfaction

Similar unnumbered unipolar visual analog scales were used to measure interest and satisfaction separately. Each end of these scales was labeled “not at all interested/satisfied” to “very interested/satisfied.” These scales ranged from 0 to 50. Previous studies have shown that this method successfully quantifies participants’ affective changes (e.g., Dijksterhuis and van Olden, 2006; Motl et al., 2018).

#### Procedures

This study was part of a larger research, and only methods relevant to the present investigation are reported here.

##### Forecasts (Time 1)

At the beginning of the Autumn term, participants needed to forecast their emotional reactions to their jobs after a busy, back-to-school month. Specifically, participants were asked to suppose, “If it is a month after autumn term beginning, you have finished your back-to-school month.” Then, they needed to complete two questions similar to past studies: participants were asked to forecast how interesting they would feel in general and how satisfied they would feel with their jobs on scales separately, ranging from (1) not at all to (50) extremely. These question formats were similar to past affective forecasting studies (Levine et al., 2012), in which participants could predict their future emotions.

##### Experience (Time 2)

Participants were asked to report their actual feelings about their jobs in a month. Participants completed the second investigation during the first day in the next month (the second month in this term). As a pair to questions in Time 1, participants were asked to report their actual feeling about their jobs: how interesting they feel in general and how satisfied they feel with their jobs right now, ranging from (1) not at all to (50) extremely.

In addition, participants filled in the blanks, reporting whether there were any special events or massive accidents in their 1-month job. Results showed that no focus events happened, indicating that their “back-to-school month” has somehow comparability.

### Results and Discussion

**FIGURE 1:**
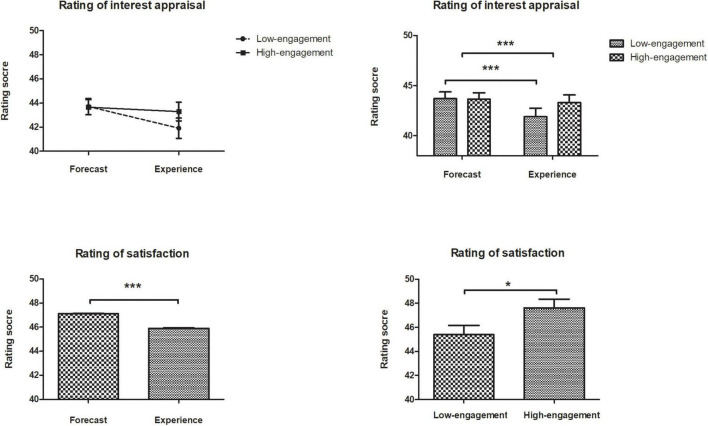
Mean rating scores in the interest appraisal and satisfaction for participants in low-/high- engagement groups.

The data were analyzed after excluding participants (*n* = 116). Figure 1 illustrates the participants’ rating scores for emotions (interest appraisals and satisfaction) in two-time points (forecasts and experience) under different groups (high-occupational-engagement and low-occupational-engagement).

#### Effect of Occupational Engagement on Kindergarten Principals’ Career Interest Appraisals

A 2 × 2 (group × time) repeated-measures analysis was conducted on the data to examine the interest changes over time, which required the Greenhouse-Geisser corrected degrees of freedom to compensate for a violation of the assumption of sphericity. In the analysis of interest appraisals, there was a significant interaction between engagement and time, *F*(1, 114) = 14.83, *p* < 0.001, η*^2^* = 0.12. Pairwise comparisons revealed significant differences between forecasts (*M* = 43.70, *SD* = 0.67) and experience (*M* = 41.91, *SD* = 0.84) in low-occupational-engagement group, *t* = −6.56, *p* < 0.001, *d* = −1.26^1^. However, the forecasts (*M* = 43.65, *SD* = 0.63) and experience (*M* = 43.29, *SD* = 0.78) did not differ in the high-occupational-engagement group, *t* = −1.39, *p* = 0.167, *d* = −0.25. In addition, there was strong evidence for differences between two-time points, *F*(1, 114) = 33.03, *p* < 0.001, η*^2^* = 0.23, participants’ forecasts (*M* = 47.12, *SD* = 0.40) significantly higher than their actual interest appraisals (*M* = 45.89, *SD* = 0.67), *t* = 1.23, *p* < 0.001, *d* = 2.11), but there was not a significant difference between two occupational engagement groups (*p* > 0.50).

As shown in Figure 1, participants in the low-occupational-engagement group produced a significant affective forecasting bias (cannot correctly predict the future experience). In contrast, participants in the high-occupational-engagement group predicted more accuracy.

#### Effect of Occupational Engagement on Kindergarten Principals’ Career Satisfaction

A 2 × 2 (group × time) repeated-measures analysis was conducted on the data to examine the emotion changes over time, which required the Greenhouse-Geisser corrected degrees of freedom to compensate for a violation of the assumption of sphericity. In the analysis of satisfaction, there was no interaction between engagement and time, *F*(1, 114) = 3.86, *p* = 0.052, η*^2^* = 0.03. However, there were significant main effects for both engagement, *F*(1, 114) = 4.52, *p* < 0.05, η*^2^* = 0.03; and time, *F*(1, 114) = 14.68, *p* < 0.001, η*^2^* = 0.11. In general, participants in the low-occupational-engagement group (*M* = 45.39, *SD* = 0.77) reported lower satisfaction than those in the high-occupational-engagement group (*M* = 47.62, *SD* = 0.72). Moreover, participants experienced lower satisfaction (*M* = 45.89, *SD* = 0.67) than they predicted (*M* = 47.12, *SD* = 0.40).

Consistent with previous findings (e.g., Wilson and Gilbert, 2003; Mata et al., 2019), both emotions, whether career interest appraisals or career satisfaction, can successfully evoke a forecasting bias (overestimation/underestimation) between two-time points (forecasts/experience). In line with the existing research, this experiment confirmed that occupational engagement plays a vital role in emotion evaluations corresponding to career context. Importantly, this experiment also revealed that occupational engagement only significantly interacted with time in interest appraisals, whereas it did not produce an interaction in satisfaction. This result indicated that the effect of occupational engagement on affective forecasting only exists in interest appraisals. Inconsistent with the finding of Werner and Milyavskaya (2018), the disappearance of the impact in satisfaction might suggest that participants’ satisfaction is a more concrete constituent of emotions. It involved more motivation and was less influenced by a latent cognitive component such as occupational engagement.

Although Experiment 1 demonstrated the effect of occupational engagement on participants’ affective forecasting and its difference pattern in two emotions, one question is still to be resolved: what makes occupational engagement influence forecasting biases of interest appraisals so much? As the hypothesis mentioned above, we try to explain this problem specifically in when features (to-be-represented information) of events are responsible for the effect of occupational engagement on affective forecasting bias. Operationally, surprisingness, one of the most influential factors for decision-making, was also believed to affect this relation. Thus, Experiment 2 discussed the relationship between surprisingness, occupational engagement, and affective forecasting bias.

## Experiment 2: Surprisingness, Occupational Engagement, and Career Interest Appraisals of College Students

Experiment 1 revealed that career interest appraisals showed a significant overestimation from forecasts to experience and indicated that participants’ occupational engagement levels affected their affective forecasting bias. These findings imply the potential role of information accessibility about to-be-predict events. However, it is hard to operate individuals’ representations of to-be-predict events, not to mention the variety and influence. Thus, Experiment 2 aimed to replicate these findings in a similar, controllable environment—preferences on vocational/educational career video (e.g., Levine et al., 2012). Experienced emotion was assessed three times during the chosen video, and these ratings were used to form a mixed interest index for each video. This index was excluded for reducing interference and enhancing the persuasion of results.

As mentioned in the “Introduction” and “Results” sections in Experiment 1, we hypothesized that interest rates would decrease over time, influenced by both occupational engagement and surprisingness, as people adopted such information to represent to-be-predict events before it happens. Before watching the chosen video, participants needed to forecast their possible interest appraisals corresponding to the TED lecture, and 1 week after watching, participants reported their recall about how attractive the chosen video was.

### Methods

#### Participants

This experiment adopted a mixed design. Participants were assigned to one of four groups by their occupational engagement levels (high vs. low) and the surprisingness of to-be-predict events (expected vs. unexpected). *A priori* power analysis indicated that 100 participants were essential to have 80% power for detecting a medium effect with the traditional α = 0.05 criterion of statistical significance (G*Power 3.1: Faul et al., 2007). Thus, 180 undergraduate students first completed OES-S (*Occupational Engagement Scale-Student*, more details seen in **Materials**). All of them were lack of real occupational experience. We then assigned 62 participants to the high-occupational-engagement group with *z*-scores greater than + 0.5 and 61 participants to the low-occupational-engagement group with *z*-scores lowed than −0.5, separately. Then, half of the participants were assigned to the expected group (*n* = 60), and half were assigned to the unexpected group (*n* = 63). As a result, a total of 123 participant cases remained for analysis (35 females and 88 males; *M*_age_ = 22.11, *SD* = 2.81). An independent-samples *t*-test confirmed that the grouping was successful for engagement. Participants in high-engagement condition (*M* = 36.58, *SD* = 2.90) would have more occupational engagement than participants in low-engagement condition (*M* = 28.39, *SD* = 4.51), *t*(121) = −11.94, *p* < 0.001, *d* = 2.16. Moreover, all participants signed a written, informed consent under the procedures and protocols approved by the human subjects’ review committee of the Northeast Normal University.

#### Materials

##### Occupational Engagement Scale-Student

The OES-S is a 9-item instrument to measure college students’ career-related experience validated by Cox et al. (2015), and it has proven reliable (α = 0.85). Internal consistency of the OES-S in the current sample (α = 0.80) suggests sufficient reliability.

##### Measurements for Interest

A similar scale with Experiment 1 was used to measure participants’ interest, which was scored with a standardized template ranging from 0 (not at all interested) to 50 (very interested).

##### Video Options

The audio-visual stimuli featured eight occupational/educational videos from the “TED talks” series. Each video was about distinct topics, ranging from 15 to 18 min. Then, these videos were summarized into a framework by a set of five different descriptions (refer to Table 1 for details). To standardize videos, we used an interest index to exclude the effect of the interest aroused by influential factors except for the topic itself (e.g., lecture skills). The interest index was reflected by a general “video quality” metric for each video, which averaged all participants’ scores for a particular video (Motl et al., 2018).

**TABLE 1:** Standardized descriptions of the eight video options.

Video	Standardized Descriptions				
	
	Field	General performance	Specific performance	Object	Goal
1	Mathematics	Study Metaphor	Formulas’ Meanings	Mathematical symbols	Understand the world by mathematical patterns
2	Market	Design Consumption Patterns	Consumption Strategies	Consumer habits	Sell products
3	Medical	Research Cancer	Experiments in patients	Anatomy	Treating Cancer
4	Politics	Studying the Currency Economy	Explaining the Currency	Bitcoin	Promotes economic development
5	Education	Reform Educational Disadvantages	Reflect on Education	Anti-test-oriented	Train children to learn initiatively
6	Engineering	Design and Development Robots	Robot design process	Robots	Create flexible robots
7	Media	Analytics Streaming	Media on Entertainment	Game Community	Build a robust interactive community
8	Psychology	Research Misconceptions	Authenticity of Testimony in Justice	Memory	Reduces memory misconstruction

##### Mood Assessment

We assessed participants’ moods by a single item with a 50-point unipolar visual analog scale, proven effective and sensitive to mood status by previous studies (e.g., Williams et al., 2010). It was used to control mood effects, a significant predictor of interest before and after participants were involved in various experimental tasks.

#### Procedures

During Session I, participants first performed a manipulation task, randomly assigned to various surprisingness groups (expected vs. unexpected). Then, the participants were instructed to choose one of the eight videos based on a framing description. After that, they needed to rate their predicted and experienced feelings before/after watching the selected video. During Session II, all participants were shown the description again and completed the third feeling rate (affective recall). Two sessions were executed 1 week apart.

##### Manipulations of Certainty Levels

###### Expected Condition

During recruitment, undergraduates in the expected condition (*n* = 60) were instructed to complete an interest rating of video materials, ultimately the same as they did in the following study. After they came into the laboratory, the instruction of the whole procedure was presented on the screen. Then, using the same scale as for interest rating, participants were asked: “How surprised will you be by this procedure?” Participants rated these appraisals on a scale from (1) *not at all surprised* to (50) *extremely surprised*.

###### Unexpected Condition

Participants in the unexpected condition (*n* = 63) were instructed to participate in a boring memory test during recruitment, quickly found counterfactual in the following procedure. After they came into the laboratory, the real aim and instruction of the whole procedure were presented on the screen. Then, the participants were asked: “How surprised will you be by this procedure?” and rated these appraisals on a scale from (1) *not at all surprised* to (50) *extremely surprised*.

###### Manipulation Check

An independent-samples t-test confirmed that participants in the unexpected condition (*M* = 34.75, *SD* = 3.31) felt more surprised than those in the expected condition (*M* = 27.46, *SD* = 5.64), *t*(121) = −8.67, *p* < 0.001, *d* = 1.58, demonstrating that the prime group was successful.

##### Forecasts (Time 1)

Participants were instructed to make predictions on an unnumbered unipolar visual analog scale. Interest items were scored with a standardized template ranging from 0 (*not at all interested*) to 50 (*extremely interested*), and previous studies have shown that this method was successful in quantifying affective changes in participants (e.g., Dijksterhuis and Nordgren, 2006; Motl et al., 2018).

##### Experience (Time 2)

Participants watched the video and rated their feelings three times to compose a relatively accurate affective experience score. They rated the feelings in 5 min after watching, 10 min after watching, and immediately after the video. Finally, there was a short interview to investigate participants’ views on the video and their reasons for choosing it.

##### Recall (Time 3)

One week later, participants picked their previous choice from the framework and then rated their feelings the third time. Fortunately, all participants successfully selected the right video and accurately recalled the core event.

### Results and Discussion

**FIGURE 2:**
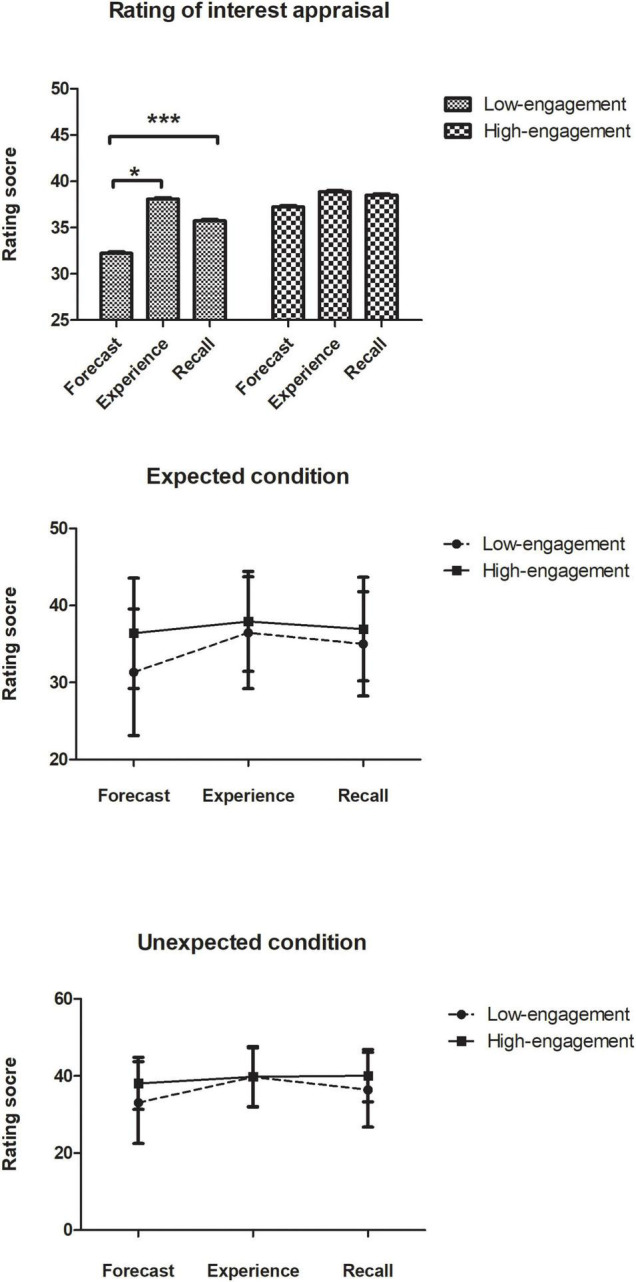
Participants’ rating scores for emotions in three-time points (forecasts, experience, and recall) under different groups.

The data were analyzed after excluding the participants (*n* = 121). Figure 2 illustrates the participants’ rating scores for emotions in three-time points (forecasts, experience, and recall) under different groups (high-engagement vs. low-engagement) by various surprisingness (high vs. low).

#### Effect of Occupational Engagement on Kindergarten Principals’ Career Interest Appraisals

A 2 × 2 × 3 (engagement × surprisingness × time) repeated-measures analysis was conducted on the data to examine the interest changes over time, which required the Greenhouse-Geisser corrected degrees of freedom to compensate for a violation of the assumption of sphericity. In the analysis of interest appraisals, there was a significant interaction between engagement and time, *F*(1.92, 228.02) = 10.06, *p* < 0.001, η*^2^* = 0.078. Pairwise comparisons revealed that in low-occupational-engagement group, the forecasts (*M* = 32.22, *SD* = 1.07) was significantly lower than experience (*M* = 35.73, *SD* = 0.97), *t* = 2.96, *p* < 0.05, *d* = 0.52, and lower than the recall (*M* = 38.09, *SD* = 0.94), *t* = 4.40, *p* < 0.001, *d* = 0.86. However, there was no such difference in the high-occupational-engagement group (all *p*-values > 0.50).

And there was strong evidence for differences between various engagement groups, *F*(1, 119) = 8.15, *p* < 0.05, η*^2^* = 0.064; different surprisingness groups, *F*(1, 119) = 4.76, *p* < 0.01, η*^2^* = 0.038; and between three-time points, *F*(1.92, 228.02) = 10.06, *p* < 0.001, η*^2^* = 0.078. Pairwise comparisons revealed a significant difference between low occupational-engagement group (*M* = 35.34, *SD* = 0.71) and high occupational-engagement group (*M* = 38.21, *SD* = 0.71), *t* = 2.85, *p* < 0.05, *d* = 0.30. And there was a significant difference between low surprisingness group (*M* = 35.68, *SD* = 0.72) and high surprisingness group (*M* = 37.87, *SD* = 0.70), *t* = 2.18, *p* < 0.05, *d* = 0.23. Moreover, there was an underestimation in affective forecasting (different between three-time points), prediction (*M* = 34.72, *SD* = 0.75) was significantly lower than experience (*M* = 37.12, *SD* = 0.69), *t* = 4.00, *p* < 0.001, *d* = 0.55; and prediction was also significantly lower than recall (*M* = 38.48, *SD* = 0.66), *t* = 2.87, *p* < 0.05, *d* = 0.35.

However, there was no evidence for an interaction between engagement and surprisingness, nor the interaction between engagement and surprisingness (all *p*-values > 0.50). In addition, there was no interaction between engagement, surprisingness, and time (all *p*-values > 0.50).

As the hypothesis mentioned above, Experiment 2 demonstrated the effect of occupational engagement on participants’ affective forecasting again and proved that the growth pattern only existed in the low occupational engagement group. Participants in the low occupational-engagement group produced a significant affective forecasting bias (a pattern of sustainable growth). In contrast, participants in the high occupational-engagement group predicted more accuracy. To our surprise, surprisingness was not effective in this relationship. This might be partly because the surprisingness has too much influence on affective rating. Not only prediction but also the experience was affected by it. These results might imply only that those features of to-be-represented events that are distinctive between the predicted phase and experience phase would have more influence on affective forecasting process.

## General Discussion

It is difficult for people to forecast their future emotions accurately, no matter how hard they try (e.g., Doré et al., 2016; Buechel et al., 2017). This study provides a more habitual context to construct participants’ occupational, emotional responses rather than general (i.e., Levine et al., 2012) and draw a picture of people’s weaknesses in affective forecasting. Based on the theoretical framework, which identified information accessibility as a contributor to forecasting bias (information accessibility, Robinson and Clore, 2002), as well as the general evaluability theory (Hsee and Zhang, 2010), we focus on the mechanisms to explain why people show bias in affective forecasting. The information accessibility in this study is the occupational engagement that people possess before forecasting. This feature was selected because of its prominence in career decision-making and affective forecasting (e.g., Motl et al., 2018). Surprisingness was another chosen feature because of its conflicting results in prior studies (e.g., Lench et al., 2019). This differentiation in patterns under various conditions is worth discussing. Taken together, these features and their influence on emotional responses across time are helpful to yield insights into the mechanisms that contribute to affective forecasting bias in daily life.

### Underestimation in Interest Appraisals but Overestimation in Satisfaction

The first question addressed by this study was whether people made forecasting bias in each career-related emotion. And if so, are they overestimations or underestimations? Substantial findings have demonstrated that people would be doomed to forecast inaccuracy: whether the target event was daily (e.g., presidential election, Hoerger and Quirk, 2010; sports competition, Mata et al., 2019) or not (e.g., non-national war or space crash, Wilson et al., 2000), people tend to make a lousy prediction; whether the emotion was happiness in general (e.g., final exam, Buehler and McFarland, 2001) or not (e.g., revenge, Lambert et al., 2014; regret, Buchanan et al., 2019; Dillard et al., 2020; curiosity, Hsee and Ruan, 2016; Ruan et al., 2018; food preference, Lee et al., 2015). Thus, we suggest that there will be a consistent wrong prediction among both career-related emotions. Two types of forecast and experienced emotions were assessed for a real-world environment–kindergarten headmasters’ career interest appraisals and career satisfaction before/during the first month in the term. Consistent with the hypotheses, participants showed a prediction mistake in forecasting their interest appraisals about the job, and there was a typical underestimation just like the inverted V-shape reported before (Motl et al., 2018).

In contrast, this tendency from forecasting to experience turned to overestimating career satisfaction. This finding is inconsistent with prior studies, and we are especially sure that there is not a procedural artifact like Levine et al. (2012) found in this investigation. We executed some analysis on individual differences variables to identify patterns that might explain why affective forecasting bias exists in interest appraisals but does not appear in career satisfaction. There was no evidence that this difference resulted from variation across individual differences (e.g., demographic distribution leads to different patterns in two emotions). Given that results, one possible explanation is that career satisfaction makes people perceive the importance of the event better. People are more cautious in making their predictions. Thus, the direction of initial bias is fixed in the overestimation, resulting in lesser accuracy toward overestimation (Lench et al., 2019). However, it was inconsistent with one prior finding on people’s attitude changes on public transportations (Werner and Milyavskaya, 2018). The intensity of satisfaction forecasting bias was found to share a similar pattern with the general aspect of people’s emotional response. Another possible reason is that career satisfaction represents participants’ attitude on a job over a longer time, which need more occupational experience to elicit a strong response, and its change always delays. Contrived selecting 1-month work practice as the time interval between prediction and experience cannot provide enough information for its change. When the short post-interview was retrieved again, there are also signs that participants ignored and underestimated their 1-month accumulations of jobs like the demonstration mentioned above: some kindergarten headmasters announced in a short sentence that they faced a minor problem or the career was not like they used to think, but they consistently put it down on coincidence and denied that the work made them upset. It is known that attribution plays a vital role in the individual prediction process (Wells and Harvey, 1978). Therefore, we inferred that if the investigation lasted for a long time, there would be another result on career satisfaction that people forecast where it may be biased. Further, some experimental manipulation is not suitable in this environment—future studies could directly examine the features of events that influence people’s emotional forecasting by diary method or interviews to achieve more information.

### Mechanisms Contributing to Bias

Our study also investigated the mechanisms that underlie affective forecasting bias on specific emotional responses. These hypotheses mechanisms were identified based on prior findings suggesting that information accessibility (occupational engagement) was the factor to determine the process of forecasting emotion, and surprise did a favor in this process (e.g., Verduyn et al., 2009, 2012; Motl et al., 2018; Lench et al., 2019). To test this assumption, we conducted a reliable labor study that experimentally manipulated participants’ occupational engagement and surprises to assess the consequences of these factors for affective forecasting biases. The results revealed that mechanisms were particularly like forecasting bias in the field study (study 1). Specifically, the level of occupational engagement led to the difference of bias pattern participants showed in forecasting how happy they would be after watching the video. Participants in the low occupational engagement condition underestimated their future emotions, and this underestimation was sustained in a week (a typical affective forecasting bias). Compared with them, participants in the high occupational engagement condition estimated more accuracy in forecasting the intensity of how happy they would feel. This result is consistent with past investigations showing that the intensity of participants’ forecasting bias is strongly related to how available the information they perceived of to-be-predicated events was (e.g., Robinson and Clore, 2002; Hsee and Zhang, 2010). Notably, the information accessibility is described as occupational engagement in this work and thus is context-specific. Motl et al. (2018) used a similar experiment design but ascribed the bias to the dual-process mechanism. In their study, a history of occupational engagement was also found particularly relevant to forecasts of the intensity of future emotion. Given these results, we suggested that participants’ history of occupational engagement, as an essential representation of information accessibility in career-related events, played a critical role in forecasting future emotions.

Surprise (a common condition or not) was believed to be crucial in emotional responses (Verduyn et al., 2009, 2012, 2013). Therefore, we hypothesized that this element influenced people’s forecasting bias. The results, however, revealed a pattern against this assumption. Although emotions shift rapidly over time and surprise affects people’s emotional responses, like those previous findings’ observations (e.g., Lench et al., 2019), the two factors turned out to be independent. We cannot find any forecasting pattern that differs between the expected and unexpected conditions. This phenomenon—that the surprise itself did not influence forecasting biases—used to be explained as the role of features that vary across conditions. However, the results of this study revealed a more nuanced potential explanation: surprise did affect responses, but the effect was relatively balanced, thus identified as an invalid factor on affective forecasting bias. Technically, in contrast to other factors such as occupational engagement, surprise merely did not change forecasting in isolation. Geng et al. (2020) have demonstrated that the inaccuracy of prediction but not inaccuracy experience promotes bias. Thus, it is reasonable to deduce that manipulation of surprise did not significantly change the magnitude of bias in forecasting interest appraisals because surprise cannot be effective in the prediction separately.

In conclusion, these findings demonstrated that the forecasting bias depends on the information available that people possess. Without enough available information, people sometimes overestimate, and sometimes underestimate their future emotions (e.g., Buechel et al., 2017; Frank et al., 2020; Kaplan et al., 2020; Villinger et al., 2020; Karl et al., 2021). Our findings indicate that people’s available information matters whatever emotion type. People can predict their future feelings more accurately with the help of available information (occupational engagement, specific in vocational context) that supports them in building up representations of events. Further, this mechanism is independent of the surprisingness of to-be-predicted events, which influences emotional responses throughout the process.

A potential limitation of this study was the hypothetical scenarios used to manipulate participants’ surprise. Previous studies selected the different risks of loss or gain to reflect people’s level of surprise and admitted that this lab setting might not make people as high-involved as the natural environment (Lench et al., 2019). We are unable to rule out this possibility as well. Although solid evidence for the effect was revealed in this investigation, future studies should further examine the role of surprise on affective forecasting by using more sensitive experimental designs (e.g., uncertainty, manipulated in the information board). Furthermore, it must be admitted that the sampling of this study is still relatively limited, and the follow-up research will improve it by expanding the sample group from the specific occupation to some others to enrich the application scope. In addition, this study also does not consider individual differences, which may be pronounced in affective forecasting bias and somehow causes or aggravate it (e.g., Spark and O’Connor, 2020; Zhang et al., 2020). The combined effect of these individual differences and information accessibility on emotional forecasting bias is mysterious and attractive.

## Data Availability Statement

The data used to support the findings of this study are available from the corresponding author upon request.

## Ethics Statement

The studies involving human participants were reviewed and approved by the human subjects review committee of Northeast Normal University. The patients/participants provided their written informed consent to participate in this study.

## Author Contributions

DL performed the material preparation, data collection and analysis, and wrote the first draft of the manuscript. All authors contributed to the study conception and design, commented on previous versions of the manuscript, read and approved the final manuscript.

## Conflict of Interest

The authors declare that the research was conducted in the absence of any commercial or financial relationships that could be construed as a potential conflict of interest.

## Publisher’s Note

All claims expressed in this article are solely those of the authors and do not necessarily represent those of their affiliated organizations, or those of the publisher, the editors and the reviewers. Any product that may be evaluated in this article, or claim that may be made by its manufacturer, is not guaranteed or endorsed by the publisher.

## Footnotes

1 Specifically, in pairwise comparisons, the results that we have reported in the manuscript are the results after Bonferroni’s correction if it is necessary.
