# Supplementary material for: Surprisingness and Occupational Engagement Influence Affective Forecasting in Career-Relevant Contexts
Source: Front Psychol. 2022 Jul 1;13:838765. doi: 10.3389/fpsyg.2022.838765 (PMC9284276; doi:10.3389/fpsyg.2022.838765)
Supplement: Supplementary file 1 [file Table_1.docx]

**Table S1.** The Descriptions Results of Participants Selections

| Video | Amounts | Ratio (%) |
| --- | --- | --- |
| 1 | 8 | 6.9 |
| 2 | 12 | 10.4 |
| 3 | 21 | 18.1 |
| 4 | 24 | 20.7 |
| 5 | 25 | 21.6 |
| 6 | 6 | 5.1 |
| 7 | 7 | 6 |
| 8 | 13 | 11.2 |
| Total | 116 | 100 |
